# Causes of Activation and Deactivation of Modified Nanogold Catalysts during Prolonged Storage and Redox Treatments

**DOI:** 10.3390/molecules21040486

**Published:** 2016-04-13

**Authors:** Ekaterina Kolobova, Yulia Kotolevich, Ekaterina Pakrieva, Grigory Mamontov, Mario H. Farías, Nina Bogdanchikova, Vicente Cortés Corberán, Alexey Pestryakov

**Affiliations:** 1 Department of Physical and Analytical Chemistry, Tomsk Polytechnic University, Tomsk 634050, Russia; ekaterina_kolobova@mail.ru (E.K.); epakrieva@mail.ru (E.P.); 2 Centro de Nanociencias y Nanotecnología, UNAM, Ensenada 22860, Mexico; Julia.Kotolevich@gmail.com (Y.K.); mario@cnyn.unam.mx (M.H.F.); nina@cnyn.unam.mx (N.B.); 3 Laboratory of Catalytic Research, Tomsk State University, Tomsk 634050, Russia; grigoriymamontov@mail.ru; 4 Institute of Catalysis and Petroleumchemistry (ICP), CSIC, 28049 Madrid, Spain; vcortes@icp.csic.es

**Keywords:** gold catalysts, support modification, CO oxidation, effect of storage, catalyst deactivation, catalyst reactivation

## Abstract

The catalytic properties of modified Au/TiO_2_ catalysts for low-temperature CO oxidation are affected by deactivation and reactivation after long-term storage and by redox treatments. The effect of these phenomena on the catalysts was studied by HRTEM, BET, SEM, FTIR CO, XPS and H_2_ TPR methods. The main cause for the deactivation and reactivation of catalytic properties is the variation in the electronic state of the supported gold, mainly, the proportion of singly charged ions Au^+^. The most active samples are those with the highest proportion of singly charged gold ions, while catalysts with a high content of trivalent gold ions are inactive at low-temperatures. Active states of gold, resistant to changes caused by the reaction process and storage conditions, can be stabilized by modification of the titanium oxide support with transition metals oxides. The catalyst modified with lanthanum oxide shows the highest stability and activity.

## 1. Introduction

The activity of catalysts based on gold nanoparticles is unique, as these catalytic systems can operate at ambient or even lower temperatures, which is crucial for a number of important industrial and environmental processes. The discovery of Haruta in the eighties of the last century that the gold deposited on metal oxides is active in the oxidation of CO at temperatures below ambient, together with works of Hutchings on hydrochlorination of acetylene on gold deposited on the carbon ushered in a new era—the era of “gold catalysis”.

Since then, an increasing number of papers and patents have been published on nanogold catalytic activity. This indicates that there is a great interest in this increasingly promising area of catalysis, both by theorists and by industrialists. Supported gold catalysts are widely used in many catalytic reactions, such as purification of hydrogen in fuel cells [1,2], organic synthesis of fine chemicals [3,4], monitoring environmental pollution [5,6,7], electrocatalysis [8,9], selective oxidation [10,11,12,13], hydrogenation reactions [3,13] and many other organic reactions [14,15,16,17,18,19,20].

Despite the unique catalytic properties of nanogold catalysts, a number of problems remain unsolved. Firstly, the effect of particle size on the activity: only gold nanoparticles smaller than 5 nm exhibit high activity; however, according to some research groups (including the authors of this work [21,22,23]) gold clusters smaller than 2 nm are the most active, while larger particles and Au^3+^ ions are virtually inactive at low temperatures. Another important and unresolved issue of gold catalysts is their rapid deactivation, both at work and during storage, which is a serious problem for their practical application. The solution to these questions would be a great contribution to the development of the theory and practice of active catalysts synthesis based on gold nanoparticles.

Among the literature works devoted to the solution of this problem, some authors [24,25,26,27,28,29,30] consider that the main cause of the catalytic activity loss of gold-containing systems is the agglomeration of gold nanoparticles during storage. The major factors influencing on the agglomeration of gold nanoparticles are light, temperature and humidity. The main idea of these works lies on the selection of optimal storage conditions under which initial size gold particles do not change. Other investigators [31,32] have linked the deactivation of gold catalysts to change in the electronic state of the deposited metal, mainly with reduction of trivalent gold to the metallic state. These studies aimed to develop methods of gold catalysts synthesis with a maximum contribution of Au^3+^ states and to study changes in the electronic state of gold under different storage conditions.

The purpose of the present research is to identify patterns of formation and stabilization of gold active states on the support surface, by varying preliminary treatment conditions and the nature of support modifying additives, as well as to identify causes of Au/TiO_2_ catalysts deactivation during storage.

## 2. Results and Discussion

Table 1 shows the results of CO oxidation on gold catalysts supported on titania, either unmodified or modified with oxides of iron, cerium and lanthanum. In the as-prepared state, catalyst with the unmodified support is the most active, while those supported on modified titanias show little activity at low temperatures.

According to literature [33], in catalysts prepared by deposition-precipitation with urea gold is on the surface a complex of trivalent gold with the urea hydrolysis products (in the as-prepared state), regardless the support nature. According to a study of the destruction process of gold precursor on the various support surface in the process of CO oxidation [34], this complex of trivalent gold is destroyed much more quickly on TiO_2_ surface than on other supports. Our results also confirm this fact: the activity of the as-prepared catalyst on unmodified support is significantly higher than that of the catalysts on modified supports. This fact explains the so high temperatures needed of 100% CO conversion on modified titania supported catalysts, because trivalent gold is catalytically inactive, as noted in our earlier works [35,36,37,38] (though in the literature there is another point of view on this matter, trivalent gold is an active participant in the catalytic process [31,32]). A sharp increase in activity of catalysts with modified titania supports observed after pre-reduction treatment: for all the catalysts 100% conversion is reached already at 30 °C. This is caused by the destruction of the complex forming several different states of gold on the support surface.

To assess the change of catalytic properties of the studied systems after storage, as-prepared samples of catalysts placed in a desiccator and kept inaccessible to light along one year were tested in CO oxidation. It turned out that in comparison with recent as-prepared samples, activity of the samples was much higher after storage, except for the unmodified support catalyst, that showed lower activity. After the pre-reduction treatment of these long term stored samples, 100% CO conversion was observed at 30 °C; this performance remained at the same level even after a series of consecutive oxidation-reduction procedures.

What is the reason for this change in catalytic properties of the samples after storage and/or pretreatment in a reducing atmosphere compared to as-prepared samples? One may hypothesize that the probable cause is the change in the electronic state of the deposited metal. To confirm this, a series of physicochemical studies was carried out.

Textural analysis showed that specific surface area of the initial titanium oxide support was reduced by 20% after modification (45 m^2^/g), regardless of the oxide modifier nature. The further gold deposition did not change the supports specific surface areas, with the exception of unmodified TiO_2_ (Table 2), which lead to equivalent surface areas for all catalysts. Elemental analysis showed also quite similar Au contents for all the catalysts.

**Figure 1:**
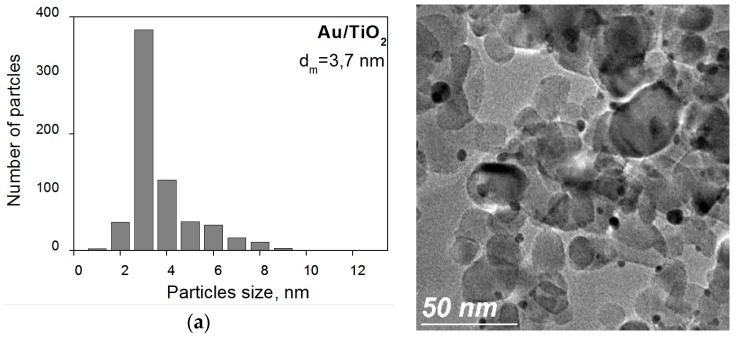

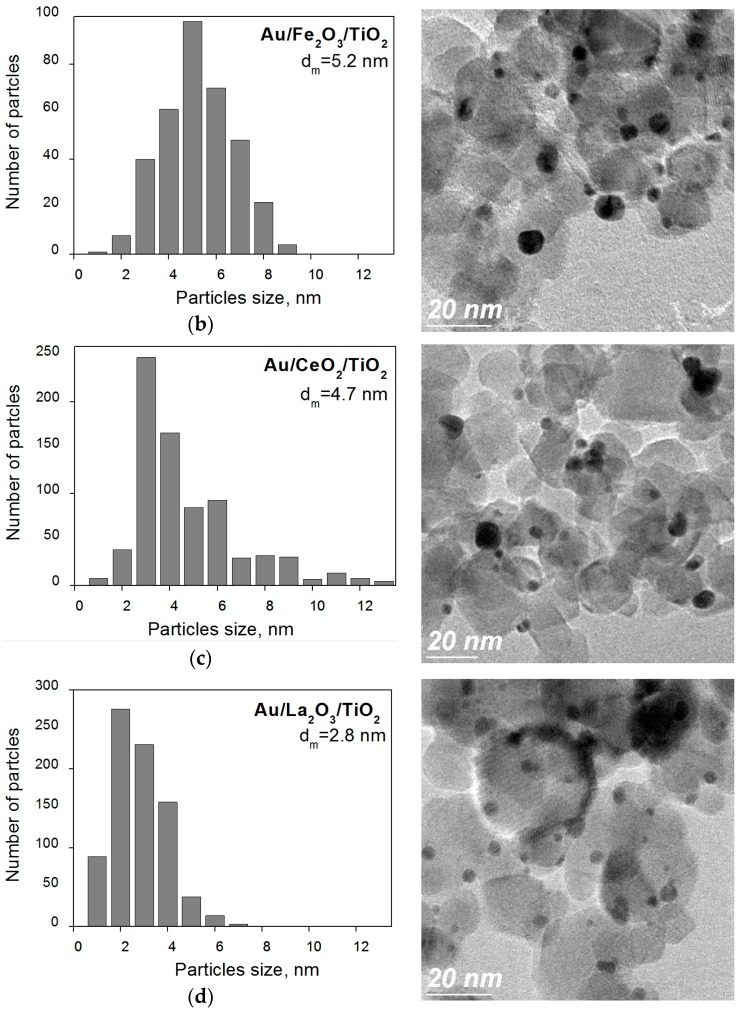
TEM images of Au/TiO_2_ (**a**) and Au/M_x_O_y_/TiO_2_ (M_x_O_y_ = La_2_O_3_, CeO_2_ or Fe_2_O_3_) (**b**–**d**) catalysts pretreated in H_2_ atmosphere at 300 °C.

Evaluation of HRTEM data showed that the average particle size (d) in the investigated catalytic systems decreases in the following order: Au/Fe_2_O_3_/TiO_2_ > Au/CeO_2_/TiO_2_ > Au/TiO_2_ > Au/La_2_O_3_/TiO_2_ (Figure 1). When comparing this order with that of catalytic activity, no direct correlation between the average particle size and activity is observed. However, one should take into account that not all metal particles visible on the micrographs, are active participants in the catalytic process, as reported previously in the literature and our previous work [21,22,23]. In addition, sintering can proceed under the influence of the high-temperature used for the reduction treatment. It has been reported that, for a number of gold-containing catalysts, only particles with size 1 nm and less are active sites of reaction [39,40,41,42,43,44,45]. Among our samples, particles with size about 1 nm were detected only for the Au/La_2_O_3_/TiO_2_ catalyst. We consider that in this case also the most active gold particles have sizes smaller than 1 nm; the larger particles are just “spectators” or much less active. Our earlier studies on Au/zeolite catalysts [46] showed that electronic state of the most likely active sites of gold in low-temperature CO oxidation are singly charged ions Au^+^, which may enter in the composition of charged clusters Au_n_^δ+^ [39,40,41,42,43,44,45]. Therefore, we carried out a number of studies of the changes of gold electronic state in the catalysts under different treatments and during storage.

**Figure 2:**
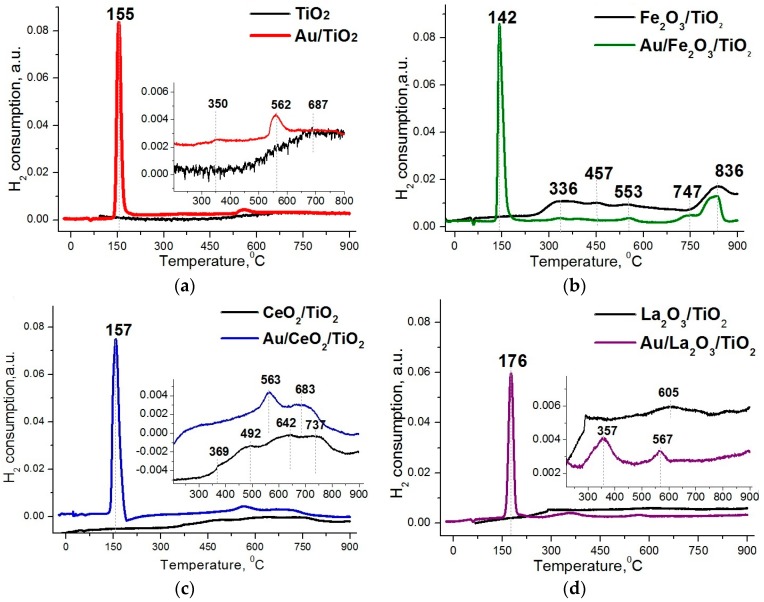
TPR profiles of Au/TiO_2_ (**a**) and Au/M_x_O_y_/TiO_2_ (M_x_O_y_ = La_2_O_3_, CeO_2_ or Fe_2_O_3_) (**b**–**d**) catalysts in as-prepared state and their supports.

Figure 2 shows the TPR profiles of supports and as-prepared Au/M_x_O_y_/TiO_2_ (M_x_O_y_ = La_2_O_3_, CeO_2_ or Fe_2_O_3_) samples. Several hydrogen consumption maxima are observed for supports in the high temperature region: for TiO_2_ (a) there is broad consumption peak with a maximum at 687 °C, related to the partial reduction of Ti^4+^ to Ti^3+^ [47,48]; for Fe_2_O_3_/TiO_2_ (b), consumption at 336–457 °C is due to the reduction of Fe_2_O_3_ to Fe_3_O_4_, and those at 553 °C and 836 °C with reduction of Fe_3_O_4_ to FeO and FeO to Fe, respectively [49]; for CeO_2_/TiO_2_ (c), consumption at 369–492 °C relates to a reduction of the surface oxygen of cerium oxide with the formation of Ce^3+^ and oxygen vacancies, and higher temperatures maxima at 642–737 °C relate to oxygen reduction of bulk cerium oxide together with the reduction of Ce^4+^ to Ce^3+^ in the structure [50,51]; for La_2_O_3_/TiO_2_ (d) a broad consumption peak with a maximum at 605 °C is observed in the high temperature region, which is probably associated with decomposition of surface lanthanum hydroxocarbonates La_2_(OH)_4_(CO_3_) [52]. For all catalyst samples the most intense hydrogen consumption is observed in the range 140–180 °C. According to literature [53] this low-temperature consumption is related to the reduction of unstable Au_2_O_3_ (ΔH_f_ = +19.3 kJ/mol), weakly interacting with the support, that probably is not an active participant in the catalytic process. In addition, for all samples, without exception, a high-temperature consumption is observed in the range of 550–570 °C. It relates to the reduction of strongly bound ionic gold, stabilized on the support surface by hydroxyl groups through the formation of centers as Ti-O-Au. All these data explain the relatively low activity of the catalysts with modified supports in as-prepared state, because highly charged gold ions Au^3+^ are catalytically inactive.

**Figure 3:**
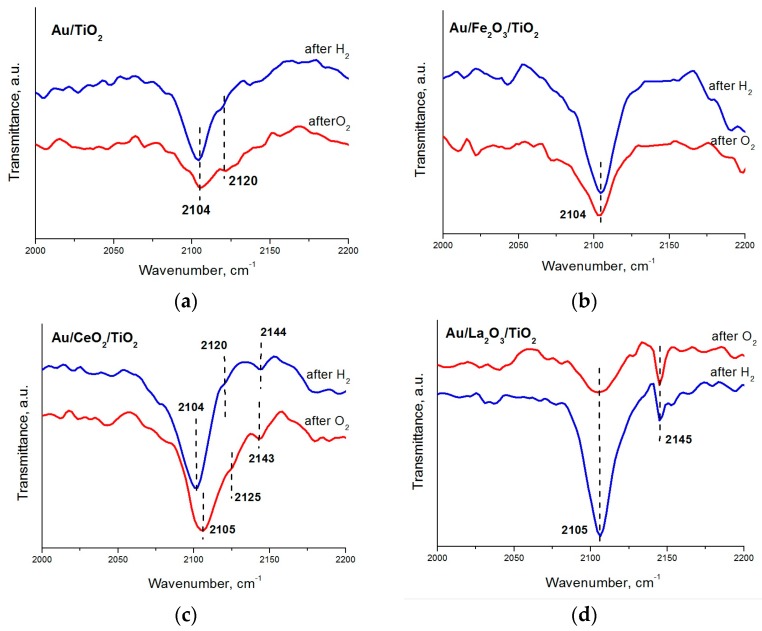
IR spectra of CO adsorbed on Au/TiO_2_ (**a**) and Au/M_x_O_y_/TiO_2_ (M_x_O_y_ = La_2_O_3_, CeO_2_ or Fe_2_O_3_) (**b**–**d**) catalysts pretreated at 300 °C for 1 h in hydrogen or oxygen atmosphere.

To assess the effects of pretreatment atmosphere on the electronic state of the gold, IR spectra were recorded for the samples reduced and then oxidized at 300 °C for 1 h. Figure 3 shows the IR spectra of CO adsorbed on Au/TiO_2_ and Au/M_x_O_y_/TiO_2_ (where M_x_O_y_ = La_2_O_3_, CeO_2_ or Fe_2_O_3_) catalysts. Regardless the pretreatment conditions, one absorption band at 2104–2105 cm^−1^, attributable to the surface carbonyls of gold atoms Au^0^-CO, is observed for all studied samples [54]. Another absorption band at 2143–2145 cm^−1^, related to the complexes of the ions Au^+^-CO, was observed only for the Au/CeO_2_/TiO_2_ and Au/La_2_O_3_/TiO_2_ samples. Moreover, the intensity of this absorption bands changed slightly with the change of pretreatment atmosphere (H_2_ and O_2_). This band corresponding to carbonyl complexes of Au^+^-CO is absent in the IR spectra of the Au/TiO_2_ and Au/Fe_2_O_3_/TiO_2_ samples. An absorption band at 2120–2125 cm^−1^, attributed to the carbonyls of reduced gold, is observed for Au/TiO_2_ and Au/CeO_2_/TiO_2_; however, its relatively high ν_CO_ wavenumber indicates that these states of gold are electron deficient (Au^δ+^), probably due to the influence of the support.

**Figure 4:**
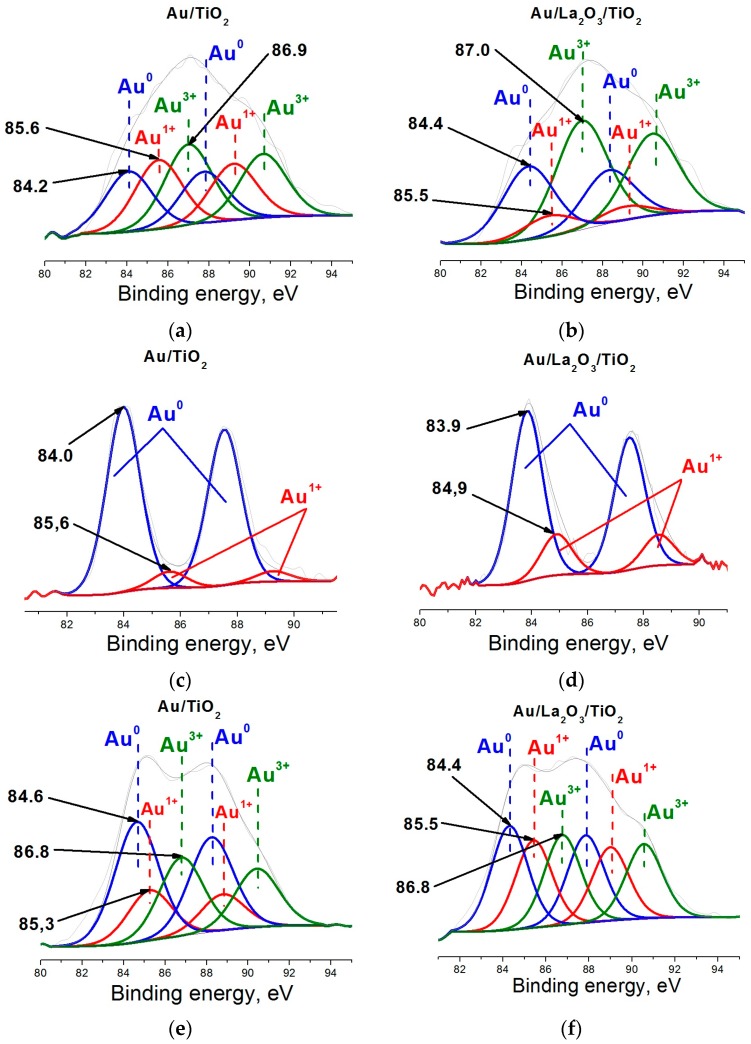
Au4f XPS spectra of Au/TiO_2_ (**a**,**c**,**e)** and Au/La_2_O_3_/TiO_2_ (**b**,**d**,**f**). Catalyts: as-prepared (**a**,**b**); after use in reaction (**c**,**d**); and after long term storage (**e**,**f**).

XPS was used for a more detailed study of changes of gold electronic state under the influence of reaction medium, and in samples after storage. In as-prepared samples with unmodified (Figure 4a) and lanthanum oxide modified (Figure 4b) supports, gold is present in three states: metallic (BE(Au4f_7/2_) = 84.2 and 84.3 eV), monovalent (BE(Au4f_7/2_) = 85.6 and 85.5 eV) and trivalent (BE(Au4f_7/2_) = 86.9 and 87.0 eV) [55].

The proportion of trivalent state (54%) in La-containing sample is significantly higher than those of monovalent (10%) and metal (36%) states while, remarkably, concentration of trivalent (39%) and monovalent (33%) gold is about the same in the unmodified support sample. After the catalytic process the proportion of singly charged ions (19%) increased almost in two times for Au/La_2_O_3_/TiO_2_ sample (Figure 4c).

In contrast, there was a significant decrease in the proportion of monovalent ions (8%) for Au/TiO_2_ sample (Figure 4d). Thus, for Au/TiO_2_ sample after the catalytic process all trivalent gold and part of monovalent gold are reduced to the metallic state (92%). This suggests a poor stabilization of monovalent ions on the surface of the unmodified support. For the Au/La_2_O_3_/TiO_2_ sample a part of trivalent gold is reduced not only to the metal, but also to monovalent state. This indicates a better stabilization of monovalent gold during the reaction in the Au/La_2_O_3_/TiO_2_ sample.

The storage process leads to redistribution in the proportions of the different gold states in comparing with as-prepared samples (Figure 4e,f). In the unmodified support sample the proportion of the metallic state (49%) increased at the cost of the significant decrease of singly charged ions (16%). These variations explain the decline in activity of this sample after storage. On the contrary, there is a significant increase in the proportion of Au^1+^ state (31%) for the stored Au/La_2_O_3_/TiO_2_ sample, compared to the as-prepared sample. At the same time, the proportion of gold in the metallic state for the sample remains at the same level. These data are in good agreement with the catalytic results. Comparison with catalytic dates shows that gold in Au^0^ state, as well as in Au^3+^ state is not an active participant in the catalytic process. But as a result of oxygen adsorption the Au^0^ metal states may be trasformed to active Au^+^. However, it is usually observed at higher temperatures, as we have shown in some previous papers [35,36,37,38].

Thus, the main cause of poor low-temperature activity of as-prepared samples, with the exception of the unmodified sample, is that the main part of the gold in these systems is present in the catalytically inactive trivalent and metal state (*cf*. TPR in Figure 2 and XPS in Figure 4b). It should be borne in mind when using of the XPS method that part of the highly charged ions of gold can be reduced during the measurement itself. Pretreatment of the catalysts in hydrogen atmosphere results in a significant increase in activity probably due to decomposition of the complex of trivalent gold forming not only metal, but also the monovalent state Au^+^ (*cf*. FTIR CO in Figure 3d), which is probably responsible for the low-temperature activity. Most of the studies on the stability of gold-titania catalysts report that these systems are very unstable and lose more than half its initial activity after prolonged storage [21,22,23,24,25,26,27,28,29]. Our research also found a decline in activity of the unmodified support sample after storage along a year. XPS data showed that in as-prepared samples, the proportion of monovalent ions (33%) in Au/TiO_2_ is much higher than in the modified support samples (10%): these data explain the high catalytic activity of the unmodified support sample in as-prepared state and evidence again that monovalent gold acts as the active sites. However, after prolonged storage a decrease in the proportion of the monovalent state was observed for this sample, together with an increase in that of the metallic state (Figure 4e). This significant transformation of monovalent gold into the metal phase also occurs after the catalytic process, and indicates poor stabilization of this state on the surface of unmodified titanium oxide (Figure 4c); this is likely to lead to loss of catalytic activity after large run times.

For the La-containing sample a quite the opposite situation was observed. In this case, the long term storage leads to an increase not only in the proportion of metal state, but also in that of monovalent state, at the expenses of trivalent gold (Figure 4f). These results in a substantial increase in activity compared with the as-prepared sample, where the proportion of monovalent gold was negligible (Figure 4b). In addition, monovalent state in this sample was much more resistant to the action of the reaction medium than in the unmodified sample (Figure 4c,d), indicating an efficient stabilization of the active sites, induced by the presence of lanthanum. According to catalytic data and FTIR CO concerning the contribution of monovalent gold ions the samples modified with iron and cerium oxides occupy an intermediate position between the modified with lanthanum oxide and unmodified samples.

## 3. Materials and Methods

### 3.1. Catalyst Preparation

Titania P25 (45 m^2^·g^−1^, nonporous, 70% anatase and 30% rutile, purity > 99.5%; Degussa, (Evonik’s Chemicals Business Area, Essen, Germany) was used as starting support. Before use, TiO_2_ was dried in air at 100 °C for at least 24 h. Oxides of Fe or Mg were used as modifiers (M). Modification of titania with molar ratio Ti/M = 40 was made by impregnation (2.5 cm^3^/g) of initial TiO_2_ with aqueous solutions of precursors Fe(NO_3_)_3_ × 9H_2_O, Ce(NO_3_)_3_ × 6H_2_O and La(NO_3_)_3_ × 6H_2_O (Aldrich, St. Louis, MO, USA). Then, impregnated products were dried at room temperature for 48 h, then at 110 °C for 4 h, and calcined at 550 °C for 4 h.

Commercial HAuCl_4_ × 3H_2_O (Aldrich) was used as gold precursor. Au/TiO_2_ and Au/M/TiO_2_ catalysts (nominal loading 4 wt. % Au, *i.e.*, 0.56 at.%) were prepared by deposition-precipitation with urea in the absence of light, following the previously reported procedure [27]. Briefly, the gold precursor (4.2 × 10^−3^ M), and the urea (0.42 M) were dissolved in 50 mL of distilled water; the initial pH of the solution was 2.4. Then, 1 g of support was added to this solution, the suspension temperature was increased to 80 °C and kept constant for 16 h under stirring. After the deposition-precipitation procedure, all samples were centrifuged, washed with distilled water four times, centrifuged again, and dried under vacuum for 2 h at 80 °C. After drying, the samples were stored at room temperature in a desiccator under vacuum, away from light, in order to prevent any alteration.

### 3.2. Sample Characterization

H_2_ TPR measurements of as-prepared samples were performed in a fixed-bed quartz reactor with an AutoChem 2950 analyzer (Micromeritics, Norcross, GA, USA). Temperature-programmed experiments were performed by heating at a rate of 10 °C·min^−1^ from 25 up to 900 °C under the reducing feed (10 vol. % of H_2_/Ar, 20 cm^3^·min^−1^). Hydrogen consumption was measured by the thermal conductivity detector.

Fourier transformed infrared (FTIR) spectra of CO adsorbed on the catalysts were recorded by using a Tensor 27 FTIR spectrometer (Bruker, Billerica, MA, USA) in transmittance mode with 4 cm^−1^ resolution. *In situ* experiments were carried out in a quartz cell with NaCl windows capable of working at temperatures from −100 to 300 °C and pressures from 10^−2^ to 760 Torr. The sample powder was pressed into disks of 13 mm diameter and weight ~20 mg. The sample was pretreated in H_2_ or O_2_ (100 Torr) at 300 °C for 1 h and then cooled down for room temperature. Then, H_2_ or O_2_ was evacuated and CO adsorption (Matheson Research grade, P^0^ = 30 Torr) were carried out. CO spectra presented in this work were obtained by subtracting the CO gas phase spectrum.

Textural properties of samples were determined from nitrogen adsorption-desorption isotherms (–196 °C) recorded with a Micromeritics TriStar 3000 apparatus (Norcross, GA, USA). Prior to experiments, samples were degassed at 300 °C in vacuum for 5 h. The adsorbed N_2_ volume was normalized to a standard temperature and pressure. The specific surface areas (S_BET_) of the samples were calculated by applying the BET method to the nitrogen adsorption data within the P/Po range 0.05–0.25.

A JEOL-5300 scanning electronic microscope (SEM) (JEOL, Tokyo, Japan) was utilized for a general sample morphology observation. Gold contents were measured by energy dispersive spectroscopy (EDS) in the same system equipped with a Kevex Superdry detector.

High resolution transmission electronic microscopy (HRTEM) studies were carried out using a JEM 2100F microscope operating (JEOL) with a 200 kV accelerating voltage. The samples were ground into a fine powder and dispersed ultrasonically in hexane at room temperature. Then, a drop of the suspension was put on a lacey carbon-coated Cu grid. At least ten representative images were taken for each sample. Particle size distribution was obtained by counting *ca*. 100 particles for each sample.

The catalysts were investigated by X-ray photoelectron spectroscopy (XPS) with a SPECS GmbH custom (SPECS Surface Nano Analysis GmbH, Berlin, Germany) made system using a PHOIBOS 150 WAL hemispherical analyzer and a non-monochromated X-Ray source. All the data were acquired using Al Kα X-rays (1486.6 eV, 200 W). A pass-energy of 50 eV, a step size of 0.1 eV, and a high-intensity lens mode were selected. The diameter of the analyzed area was 3 mm. Charging shifts were referenced against adventitious carbon (C 1s at binding energy (BE) 284.5 eV). The pressure in the analysis chamber was maintained lower than 1 × 10^−8^ mbar. Catalysts were mounted on a sample holder and kept overnight in high vacuum in the preparation chamber before they were transferred to the analysis chamber of the spectrometer. Energy regions were selected after a general survey and scanned with several sweeps until a good signal-to-noise ratio was observed. The accuracy of the binding energy (BE) values was ±0.1 eV. Spectra are presented without smoothing or background subtraction, with intensity in counts-per-second (CPS). Peak intensities were estimated by calculating the integral of each peak after subtracting a Shirley type background and fitting the experimental peak to a combination of Lorentzian/Gaussian lines with a 30/70 proportion, considering the spin-orbit 4f_7/2_ and 4f_5/2_ doublet with a 4:3 intensity ratio and the same width on all lines.

### 3.3. Catalytic Testing

The activity of the catalysts in CO oxidation was studied at atmospheric pressure in a flow reactor (internal diameter 9 mm) with a fixed bed of the catalyst (particle size from 0.15 to 0.2 mm, mass 0.5 g), using a reactant gas mixture: 1 vol. % CO + 1 vol. % O_2_ in Ar (total flow rate 200 mL/min) in the temperature range from 25 to 305 °C.

To study the effect of atmosphere pretreatment on the catalyst activity, prior to CO oxidation test the samples were treated in different gas mixtures: reductive (15 vol. % H_2_ in Ar) and consecutive oxidative (15 vol. % O_2_ in Ar), with a total flow rate of 200 mL/min, at 300 °C for 1 h; then the reactor was cooled to 25 °C and CO oxidation test was carried out.

The reaction mixture was analyzed on a CHROMOS GC-1000 gas chromatograph (CHROMOS, Dzerzhinsk, Russia), provided with a TCD, and using two separate packed columns filled with CaA (to analyze oxygen and hydrogen) and AG-3 sorbent (to analyze both carbon oxides), respectively, and He as carrier gas. The catalytic activity of the samples was evaluated by the magnitude of CO conversion degree by the following Equation (1):

(1) $$X_{\text{CO}}\left(\text{\%}\right)=\frac{C_{i}-C_{f}}{C_{i}}\times 100$$

where, C_i_ and C_f_ are the initial and the final CO concentrations, respectively.

## 4. Conclusions

- Variations in the electronic state of gold are the main cause of the deactivation and activation of the catalysts during redox treatments and after storage.
- The most active catalysts are those with the highest proportion of singly charged ions of gold. Au^+^ ions seem to be the active sites of the studied catalysts for low-temperature CO oxidation.
- Catalysts with a high content of trivalent gold ions Au^3+^ and Au^0^ are inactive at low reaction temperatures.
- The active states of gold in gold-titania catalysts, resistant to the reaction medium and storage conditions, can be stabilized by modification of titanium oxide support with oxides of transition metals.
- Titania modified with lanthanum oxide provides the highest stability and activity after prolonged storage for nanogold catalysts.

## Figures and Tables

**Table 1:** Temperature of 100% CO conversion for different catalyst states as a function on the nature of support modifying additives.

Sample	Temperature for 100% CO Conversion, °C				
	1 *	2 *	3 *	4 *	5 *
Au/TiO_2_	30	30	80	30	30
Au/Fe_2_O_3_/TiO_2_	305	30	80	30	30
Au/CeO_2_/TiO_2_	230	30	80	30	30
Au/La_2_O_3_/TiO_2_	215	30	30	30	30

1 *—as-prepared; 2 *—recently prepared after H_2_ (300 °C, 1 h) treatment; 3 *—after storage (about 1 year); 4 *—after long storage and H_2_ (300 °C, 1 h) treatment; 5 *—after long storage, H_2_ (300 °C, 1 h) and consecutive O_2_ (300 °C, 1 h) treatments.

**Table 2:** Textural properties of supports and catalysts, and gold content in the studied catalysts.

Sample	S_BET_, m^2^/g		EDX
	Support	Catalyst	Au Content, wt. %
Au/TiO_2_	55.5	45.5	4.5
Au/La_2_O_3_/TiO_2_	45.3	45.2	3.6
Au/CeO_2_/TiO_2_	43.4	46.6	3.5
Au/Fe_2_O_3_/TiO_2_	45.5	44.2	4.2

## Abbreviations

The following abbreviations are used in this manuscript:

BE	binding energy
BET	Brunauer-Emmett-Teller, method of specific surface area measurement
EDS	energy dispersive spectroscopy
HRTEM	high resolution transmission electron microscopy
SEM	scanning electronic microscope
TPR	temperature-programmed reduction
XPS	X-ray photoelectron spectroscopy
FTIR CO	Fourier transformed infrared (FTIR) spectrometry of adsorbed CO

## References

[1] Park E.D., Lee D., Lee H.C. (2009). Recent progress in selective CO removal in a H_2_-rich stream. Catal. Today.

[2] Manzolia M., Avgouropoulos G., Tabakova T., Papavasiliou J., Ioannides T., Boccuzzi F. (2008). Preferential CO oxidation in H_2_-rich gas mixtures over Au/doped ceria catalysts. Catal. Today.

[3] McEwan L., Julius M., Roberts S., Fletcher J.C.Q. (2010). A review of the use of gold catalysts in selective hydrogenation reactions. Gold Bull..

[4] Du X.L., He L., Zhao S., Liu Y.M., Cao Y., He H.Y., Fan K.N. (2011). Hydrogen-independent reductive transformation of carbohydrate biomass into γ-valerolactone and pyrrolidone derivatives with supported gold catalysts. Angew. Chem. Int. Ed..

[5] Wong M.S., Alvarez P.J.J., Fang Y.-L., Akёcin N., Nutt M.O., Miller J.T., Heck K.N. (2009). Cleaner water using bimetallic nanoparticle catalysts. J. Chem. Technol. Biotechnol..

[6] Hernández W.Y., Romero-Sarria F., Centeno M.A., Odriozola J.A. (2010). *In situ* characterization of the dynamic gold support interaction over ceria modified Eu^3+^. Influence of the oxygen vacancies on the CO oxidation reaction. J. Phys. Chem. C.

[7] Reina T.R., Ivanova S., Domínguez M.I., Centeno M.A., Odriozola J.A. (2012). Sub-ambient CO oxidation over Au/MO_x_/CeO_2_-Al_2_O_3_ (M = Zn or Fe). Appl. Catal. A Gen..

[8] Miah M.R., Ohsaka T. (2009). Electrocatalysis of underpotential deposited tin-adatoms-modified gold electrodes toward oxygen reduction reaction in acidic media. J. Electrochem. Soc..

[9] Murray R.W. (2008). Nanoelectrochemistry: Metal nanoparticles, nanoelectrodes, and nanopores. Chem. Rev..

[10] Della Pina C., Falletta E., Prati L., Rossi M. (2008). Selective oxidation using gold. Chem. Soc. Rev..

[11] Gong J., Mullins C.B. (2009). Surface science investigations of oxidative chemistry on gold. Acc. Chem. Res..

[12] Turner M., Golovko V.B., Vaughan O.P.H., Abdulkin P.A., Berenguer-Murcia M.S., Tikhov M.S., Johnson B.F.G., Lambert R.M. (2008). Selective oxidation with dioxygen by gold nanoparticle catalysts derived from 55-atom clusters. Nature.

[13] Yang X.F., Wang A.Q., Wang Y.L., Zhang T., Li J. (2010). Unusual selectivity of gold catalysts for hydrogenation of 1,3-butadiene toward cis-2-butene: A joint experimental and theoretical investigation. J. Phys. Chem..

[14] Bertelsen S., Jirgensen K.A. (2009). Organocatalysis—After the gold rush. Chem. Soc. Rev..

[15] Vinod C.P., Wilson K., Lee A.F. (2011). Recent advances in the heterogeneously catalysed aerobic selective oxidation of alcohols. J. Chem. Technol. Biotechnol..

[16] Katryniok B., Kimura H., Skrzynska E., Girardon J.-S., Fongarland P., Capron M., Ducoulombier R., Mimura N., Paul S., Dumeignil F. (2011). Selective catalytic oxidation of glycerol: Perspectives for high value chemicals. Green Chem..

[17] Abu Sohel S.M., Liu R.-S. (2009). Carbocyclisation of alkynes with external nucleophiles catalysed by gold. Platinum and other electrophilic metals. Chem. Soc. Rev..

[18] Takei T., Okuda I., Bando K.K., Akita T., Haruta M. (2010). Gold clusters supported on La(OH)_3_ for CO oxidation at 193 K. Chem. Phys. Lett..

[19] Date M., Okumura M., Tsubota S., Haruta M. (2004). Vital role of moisture in the catalytic activity of supported gold nanoparticles. Angew. Chem. Int. Ed..

[20] Han Y.F., Zhong Z.Y., Ramesh K., Chen F.X., Chen L., White T., Tay Q., Yaakub S.N., Wang Z. (2007). Au promotional effects on the synthesis of H_2_O_2_ directly from H_2_ and O_2_ on supported Pd-Au alloy catalysts. J. Phys. Chem. C.

[21] Pestryakov A.N., Lunin V.V., Bogdanchikova N., Temkin O.N., Smolentseva E. (2013). Active states of gold in small and big metal particles in CO and methanol selective oxidation. Fuel.

[22] Bogdanchikova N., Pestryakov A.N., Tuzovskaya I., Zepeda T.A., Farias M.H., Tiznado H., Martynyuk O.A. (2013). Effect of redox treatments on activation and deactivation of gold nanospecies supported on mesoporous silica in co oxidation. Fuel.

[23] Bogdanchikova N., Pestryakov A., Farias M.H., Diaz J.A., Avalos M., Navarrete J. (2008). Formation of TEM- and XRD-undetectable gold clusters accompanying big gold particles on TiO_2_-SiO_2_ supports. Solid State Sci..

[24] Akita T., Lu P., Ichikawa S., Tanaka K., Haruta M. (2001). Analytical TEM study on the dispersion of Au nanoparticles in Au/TiO_2_ catalyst prepared under various temperatures. Surf. Interface Anal..

[25] Schumacher B., Plzak V., Kinne M., Behm R.J. (2003). Highly active Au/TiO_2_ catalysts for low-temperature CO oxidation: Preparation, conditioning and stability. Catal. Lett.

[26] Moreau F., Bond G.C. (2006). Gold on titania catalysts, influence of some physicochemical parameters on the activity and stability for the oxidation of carbon monoxide. Appl. Catal. A.

[27] Date M., Ichihashi Y., Yamashita T., Chiorino A., Boccuzzi F., Haruta M. (2002). Performance of Au/TiO_2_ catalyst under ambient conditions. Catal. Today.

[28] Lee W.S., Wan B.Z., Kuo C.N., Lee W.C., Cheng S. (2007). Maintaining catalytic activity of Au/TiO_2_ during the storage at room temperature. Catal. Commun..

[29] Haruta M., Tsubota S., Kobayashi T., Kageyama H., Genet M.J., Delmon B. (1993). Low-temperature oxidation of CO over gold supported on TiO_2_, α-Fe_2_O_3_, and Co_3_O_4_. J. Catal..

[30] Zanella R., Louis C. (2005). Influence of the conditions of thermal treatments and of storage on the size of the gold particles in Au/TiO_2_ samples. Catal. Today.

[31] Jia M., Li X., Zhaorigetu, Shen Y., Li Y. (2011). Activity and deactivation behavior of Au/LaMnO_3_ catalysts for CO oxidation. J. Rare Earths.

[32] Wu Y., Sun K.Q., Yu J., Xu B.Q. (2008). A key to the storage stability of Au/TiO_2_ catalyst. Phys. Chem. Chem. Phys..

[33] Zanella R., Delannoy L., Louis C. (2005). Mechanism of deposition of gold precursors onto TiO_2_ during the preparation by cation adsorption and deposition-precipitation with NaOH and urea. Appl. Catal. A Gen..

[34] Delannoy L., Weiher N., Tsapatsaris N., Beesley A.M., Nchari L., Schroeder S.L.M., Louis C. (2007). Reducibility of supported gold (III) precursors: Influence of the metal oxide support and consequences for CO oxidation activity. Top. Catal..

[35] Pestryakov A.N., Bogdanchikova N., Simakov A., Tuzovskaya I., Jentoft F., Farias M., Diaz A. (2007). Catalytically active gold clusters and nanoparticles for CO oxidation. Surf. Sci..

[36] Smolentseva E., Bogdanchikova N., Simakov A., Pestryakov A., Avalos M., Farias M.H., Tompos A., Gurin V. (2007). Catalytic activity of gold nanoparticles incorporated into modified zeolites. J. Nanosci. Nanotechnol..

[37] Pestryakov A., Tuzovskaya I., Smolentseva E., Bogdanchikova N., Jentoft F.C., Knop-Gericke A. (2005). Formation of gold nanoparticles in zeolites. Int. J. Modern Phys. B.

[38] Pestryakov A.N., Lunin V.V., Kharlanov A.N., Bogdanchikova N.E., Tuzovskaya I.V. (2003). Electronic state of gold in supported clusters. Eur. Phys. J. D.

[39] Hashmi A.S.K., Hutchings G.J. (2006). Gold catalysis. Angew. Chem. Int. Ed..

[40] Bond G.C., Louis C., Thompson D.T. (2006). Catalysis by Gold.

[41] Qu Z., Huang W., Cheng M., Bao X. (2005). Restructuring and redispersion of silver on SiO_2_ under oxidizing/reducing atmospheres and its activity toward CO oxidation. J. Phys. Chem. B.

[42] Furusawa T., Seshan K., Lercher J.A., Lefferts L., Aika K. (2002). Selective reduction of NO to N_2_ in the presence of oxygen over supported silver catalysts. Appl. Catal. B Environ..

[43] Herzing A.A., Kiely C.J., Carley A.F., Landon P., Hutchings G.J. (2008). Identification of active gold nanoclusters on iron oxide supports for CO oxidation. Science.

[44] Martynyuk O., Kotolevich Y., Vélez R., Cabrera Ortega J.E., Tiznado H., Zepeda T., Mota-Morales J.D., Pestryakov A., Bogdanchikova N. (2016). On the High Sensitivity of the Electronic States of 1 nm Gold Particles to Pretreatments and Modifiers. Molecules.

[45] Deng W., De Jesus J., Saltsburg H., Flytzani-Stephanopoulos M. (2005). Low-content gold-ceria catalysts for the water-gas shift and preferential CO oxidation reactions. Appl. Catal. A Gen..

[46] Simakov A., Tuzovskaya I., Pestryakov A., Bogdanchikova N., Gurin V., Avalos M., Farías M.H. (2007). On the nature of active gold species in zeolites in CO oxidation. Appl. Catal. A.

[47] Parida K.M., Sahu N., Mohapatra P., Scurrell M.S. (2010). Low temperature CO oxidation over gold supported mesoporous Fe–TiO_2_. J. Mol. Cat. A Chem..

[48] Lenzi G.G., Fávero C.V.B., Colpini L.M.S., Bernabe H., Baesso M.L., Specchia S., Santos O.A.A. (2011). Photocatalytic reduction of Hg(II) on TiO_2_ and Ag/TiO_2_ prepared by the sol–gel and impregnation methods. Desalinations.

[49] Biabani-Ravandi A., Rezaei M., Fattah Z. (2013). Catalytic Performance of Ag/Fe_2_O_3_ for the low temperature oxidation of carbon monoxide. Chem. Eng. J..

[50] Qu Z., Yu F., Zhang X., Wang Y., Gao J. (2013). Support effects on the structure and catalytic activity of mesoporous Ag/CeO_2_ catalysts for CO oxidation. Chem. Eng. J..

[51] Li S., Zhu H., Qin Z., Wang G., Zhang Y., Wu Z., Li Z., Chen G., Dong W., Wu Z. (2014). Morphologic effects of nano CeO_2_–TiO_2_ on the performance of Au/CeO_2_–TiO_2_ catalysts in low-temperature CO oxidation. Appl. Catal. B Environ..

[52] Wang Y., Liang S., Cao A., Thompson R.L., Vesera G. (2010). Au-mixed lanthanum/cerium oxide catalysts for water gas shift. Appl. Catal. B Environ..

[53] Oros-Ruiz S., Zanella R., López R., Hernández-Gordillo A., Gómez R. (2013). Photocatalytic hydrogen production by water/methanol decomposition using Au/TiO_2_ prepared by deposition–precipitation with urea. J. Hazard. Mater..

[54] Penkova A., Chakarova K., Laguna O.H., Hadjiivanov K., Romero Saria F., Centeno M.A., Odriozola J.A. (2009). Redox chemistry of gold in a Au/FeO_x_/CeO_2_ CO oxidation catalyst. Catal. Commun..

[55] Casaletto M.P., Longo A., Martorana A., Prestianni A., Venezia A.M. (2006). XPS study of supported gold catalysts: The role of Au^0^ and Au^δ+^ species as active sites. Surf. Interface Anal..

